# Computational interactions between decision and emotion

**DOI:** 10.1186/1471-2202-16-S1-P244

**Published:** 2015-12-18

**Authors:** Nicoladie D Tam

**Affiliations:** 1 Department of Biological Sciences, University of North Texas, Denton, TX 76203, USA

## Introduction

Decision is often influenced by emotions, such that the decision can often be biased by the emotional influences, such as happiness, sadness, fear and anger. Previous experimental studies in human subjects have shown that decisions are often related to emotional levels [1,2]. It is also often assumed that decision is influenced by emotion, but there is evidence that decision can also influence emotions. Thus, the interrelationship between decision and emotion requires in-depth re-examination to determine the interactions between decision and emotion.

## Methods:

A computational model of decision-making relative to emotion is derived based on the experimental evidence of decision in relation to emotion in human subjects. Using the classical behavioral economic experimental Ultimatum Game paradigm [3] that elicits the interrelationship between decision and emotion in human subjects [4-7], a computational model of decision is derived based on the shifting of the threshold in the stimulus-response function of emotional responses.

## Results:

Using the quantitative analysis, the emotional stimulus-response function was derived based on the disparity between the desirable predicted outcome and the actual outcome in the real world for happy [4], sad [6], angry [5], jealous [7] emotions and for fairness perception [8], using the optimizations in survival functions [9,10]. The relationships between decision and emotions were also established for happy [1] emotion, and for fairness [11] experimentally, and the interrelationship between decision and fairness was derived computationally [12].

Extending the result to emotions, let **e **be the vector representing the emotional intensity, **d **be the vector representing the disparity between the predicted outcome and actual outcome, then the emotional stimulus-response function is given by: **e **= *kf*(**d**) + *b *where *f*(·) represents a nonlinear function, and the coefficient *k *is the emotional sensitivity and *b *is the emotional baseline level. Let **x **be the vector representing the decision (where **x **= 1 represents a yes decision, and **x **= -1 represents a no decision), then the decision threshold can be given by: $x=\{\begin{array}{c} 1,\text{ ; ; ; ;if ;}kf(d)+b\geq \theta \\ -1,\text{ ; ;otherwise ; ; ; ; ; ; ; ;} \end{array}$ where is the decision threshold, representing the dependence of decision on emotion.

## Discussion:

The interrelationship between decision and emotion can be derived based on the threshold crossing of the emotional intensity level, in which an emotional bias in either the emotional baseline or the emotional sensitivity can cause a change in decision. The decision is based on the threshold crossing of the emotional stimulus-response function, such that it is a continuum in altering the decision-making process by the shifting of emotional bias in either emotional baseline or sensitivity.
